# Short-Chain Fatty Acid Levels after Fecal Microbiota Transplantation in a Pediatric Cohort with Recurrent *Clostridioides difficile* Infection

**DOI:** 10.3390/metabo13101039

**Published:** 2023-09-27

**Authors:** Alison T. Jess, George Hany Eskander, My H. Vu, Sonia Michail

**Affiliations:** 1 Keck School of Medicine, University of Southern California, Los Angeles, CA 90089, USA; ajess@usc.edu; 2 School of Medicine & Health Sciences, George Washington University, Washington, DC 20052, USA; georgeeskander@gwu.edu; 3 Biostatistics Core, The Saban Research Institute, Children’s Hospital Los Angeles, Los Angeles, CA 90027, USA; myvu@chla.usc.edu; 4 Division of Gastroenterology, Children’s Hospital of Los Angeles, Los Angeles, CA 90027, USA

**Keywords:** *C. difficile*, SCFA, pediatric, FMT, propionate, isovalerate, *Clostridium difficile*, short-chain fatty acids

## Abstract

Though antibiotics are the mainstay treatment for *Clostridioides difficile*, a large population of individuals infected will experience recurrence. In turn, fecal microbiota transplantation (FMT) has emerged as a promising treatment for recurrent *C. difficile* infection (rCDI). Mechanistically, by providing a healthy, diverse flora to the infected individual, FMT “resets” the underlying gut microbiome dysbiosis associated with rCDI. A proposed mechanism through which this occurs is via microbiome metabolites such as short-chain fatty acids (SCFAs); however, this has not been previously studied in pediatric patients. Using mass spectroscopy, we quantified pre- and post-transplant levels of acetate, isovalerate, butyrate, formate, and propionate in pediatric patients diagnosed with rCDI (*n* = 9). We compared pre- and post-transplant levels within the rCDI cohort at 1, 3, 6, and 12 months post-transplant and correlated these levels with healthy controls (*n* = 19). We witnessed a significant difference in the combined SCFA levels and the individual levels of acetate, butyrate, isovalerate, and propionate in the pre-treatment rCDI cohort compared to the healthy controls. In addition, there was a significant increase in combined SCFA levels at 12 months post-transplant within the rCDI group compared to that of their pre-transplant levels, and, more specifically, acetate, propionate, and isovalerate increased from pre-transplant to 12 months post-transplant. The longitudinal aspect of this study allowed us to identify mechanisms that contribute to the durability of responses to FMT, as well as characterize the unique patterns of short-chain fatty acid level recovery in rCDI pediatric patients.

## 1. Introduction

*Clostridioides difficile*, clinically known as *Clostridium difficile*, is an anaerobic, gram-positive, spore-forming, toxin-producing bacillus. *C. difficile* colonizes the large intestine and causes disease predominantly through its production of cytoskeletal-modifying exotoxins. These toxins mediate disease by triggering colonocyte death, causing the loss of intestinal barrier function, and provoking neutrophilic colitis [1]. *C. difficile* is transmitted through the fecal–oral ingestion of spores. Although generally associated with nosocomial transmission, the incidence of *C. difficile* infection in pediatric populations has increased dramatically over the past three decades in hospital, community, and outpatient settings [2].

*C. difficile* infection (CDI) is thought to be the result of gastrointestinal dysbiosis that disrupts the resident microbiota, thereby creating an environment that is either favorable to colonization by *C. difficile* spores or to overgrowth by indigenous *C. difficile* residents [3]. The typical insult is antibiotic use, though other factors such as advanced age, prolonged hospitalization, proton-pump inhibitor use, an immunocompromised state, and other medical comorbidities have been associated with an increased risk of *C. difficile* [2]. In the pediatric population, *C. difficile* infection is associated with prior antibiotic and proton-pump inhibitor use [4].

The conventional first-line treatment for *C. difficile* in both adult and pediatric populations is antibiotic therapy, specifically metronidazole or vancomycin. Despite this, there is evidence that antibiotics can be ineffective in resolving recurrent *Clostridioides difficile* infection, and instead could potentially be counteractive by perpetuating underlying dysbiosis [5,6].

The recurrence rate of *C. difficile* in the adult population after metronidazole or vancomycin is estimated to be 20.2% and 18.4%, respectively [7]. As a result, fecal microbiota transplantation (FMT) has emerged as a recommended treatment by the Infectious Disease Society of America (ISDA) and the Society for Healthcare Epidemiology of America (SHEA) for patients with multiple recurrences of CDI who have failed appropriate antibiotic therapy [2]. This recommendation is supported by data that estimate that the cure rate of FMT for recurrent *C. difficile* infection (rCDI) is between 89.1 and 89.7% (unweighted pooled resolution rate to weighted pooled resolution rate, respectively; 95% CI 84–93%) [8].

Given that the rate of recurrent *C. difficile* infection in the pediatric population is similar to that of adults (20–30%) [9], FMT has also emerged in this population as a highly effective alternative to antibiotic therapy [10]. A recent retrospective study of 372 patients from 11 months to 23 years old who underwent FMT reported that it was 81% effective with a 4.7% rate of serious adverse events during the 3-month follow-up period [11].

Though studies in pediatric rCDI patients have shown a post-FMT increase in bacterial diversity in its recipients up to 6 months after transplant [12,13], the mechanism through which this healing occurs is largely unknown. In adults, short-chain fatty acids (SCFAs), a byproduct of microbial metabolism, have been implicated as a potential mediator in the post-transplant restoration of microbial diversity and richness in rCDI patients [14,15].

In short, SCFAs are a subset of fatty acids that are byproducts of the microbial fermentation of partially and nondigestible carbohydrates, more specifically polysaccharides, or, in the case of valeric acid, isovaleric acid, and isobutyric acid, amino acid derivatives [16]. The butyrate production pathway involves the arrangement of acetyl CoA, which is converted to acetoacetyl CoA [17]. The acetoacetyl CoA is then converted into butyryl CoA through two different enzymes, which makes butyrate [17]. Propionic acid is formed from two products, either succinate or 1,2 propanediol, which are products of broken-down carbohydrates [18]. Acetate and formate form through CO_2_ reduction reactions in the Wood–Ljungdahl pathway [19]. Isovaleric acid is formed through amino acid catabolism as an end product of oxidative phosphorylation [20].

We chose to focus our project on the overall production of SCFAs rather than delving into individual pathways. SCFAs are able to interact with the human intestinal lumen as a fuel source, through G-protein coupling receptor (GCPR) pathways and histone deacetylation inhibition [21]. Through these interactions, SCFAs have widespread systemic effects. Although our complete understanding of SCFAs’ role in the human body is yet to be actualized, prior studies have implicated various roles, including altering chemotaxis and phagocytosis [21,22]; inducing reactive oxygen species; modifying cell function and proliferation; altering gut integrity [23]; and acting as anti-inflammatory [21,24], antitumorigenic, and antimicrobial molecules [21]. In this study, we evaluated the impact of FMT on bacterial producers of SCFAs in pediatric patients with rCDI over a 12-month post-op period. Due to the introduction of diverse microbiota from FMT, we hypothesized that levels of SCFAs would increase post-op in our study population to match the profiles of healthy controls.

## 2. Materials and Methods

This study was approved by the Children’s Hospital of Los Angeles’ institutional review board as protocol number CCI-11-000148, and written informed consent was obtained from all participants prior to the initiation of study activities. To study the changes that occur in pediatric patients with rCDI treated with FMT, we conducted a longitudinal, retrospective, cohort study at Children’s Hospital Los Angeles and its referral sites.

We included a total of 9 subjects with rCDI who underwent fecal microbiota transplantation between January 2014 and December 2015 with ages ranging from 2 to 17 and 19 healthy controls with ages ranging from 2 to 16.

### 2.1. Pediatric Subjects with Recurrent Clostridioides Difficile Infection Who Responded to Fecal Microbiota Transplantation

Patients between the ages of 2–17 years old with recurrent *C. difficile* infection were included in this study. Recipients of FMT had at least two documented *C. difficile* infections associated with diarrhea (>3 loose stools/day). Recipients were also without any immune deficiencies, chronic systemic diseases, allergies to oral vancomycin, concurrent infections requiring antibiotics, exposure to probiotics within 3 weeks of FMT, known food allergies, or pregnancies. We excluded anyone with evidence of *Clostridioides difficile* colonization without gastrointestinal symptoms to suggest colitis (e.g., diarrhea, rectal bleeding).

Following informed consent, FMT recipients were asked to collect a baseline stool sample. This was processed according to the methods detailed in stool collection, below. Prior to the procedure, recipients underwent a standard colonoscopy prep using polyethylene glycol. The fecal matter from the donors was prepared as detailed below (Method of fecal microbiota transplantation). A standard colonoscopy was performed and the FMT material was administered via colonoscope starting as close to the ileocecal valve as possible.

Subjects completed follow-up visits at 1 month, 3 months, 6 months, and 12 months post-FMT. At these visits, stool samples were collected, as described below. In addition, at each visit, an evaluation of colitis-associated symptoms (abdominal pain, diarrhea, rectal bleeding), an assessment of dietary change, and a *C. difficile* polymerase chain reaction (PCR) stool test were conducted.

Study failure was defined as a positive *C. difficile* PCR test in the presence of diarrhea (>3 loose stools/day) at least ten days after intervention and up to the twelve months of the study.

### 2.2. Healthy Controls

The healthy controls were between the ages of 2 and 16, a similar age range to our study population, within the 5th–85th weight percentile for age. Subjects were excluded if they had a chronic illness or were on any medications.

Following informed consent, healthy controls completed one study visit. At this time, stool samples were collected, as described below.

### 2.3. Universal Donors

While a pediatric donor may be more appropriate for a pediatric study, this would deviate from previously published protocols. Therefore, two universal donors were recruited to the study who identified as being healthy adults less than 65 years old. The selection criteria were based on the methods described in Bakken et al. [25]. Briefly, the universal donors met the following criteria: had a body mass of 18.5 and 25, were without any chronic illness, were on regular diets, and had not taken any medications (prescription, over-the-counter, probiotics) for a least three months prior to fecal donation. The donors had negative serum HIV, Hepatitis A, B, and C, and syphilis tests. Their stool samples were negative for culture, ova and parasites, *C. difficile*, giardia, and cryptosporidium. Though two donors were recruited, samples were only used from one donor who was 18 years of age. The donor samples had a similar SCFA profile as the healthy children cohort.

### 2.4. Stool Collection

Stool samples from our rCDI cohort were collected at baseline, 1 month post-FMT (M1), 3 months post-FMT (M3), 6 months post-FMT (M6), and 12 months post-FMT (M12). Our healthy controls collected stool samples at their initial baseline appointment. At these timepoints, stool was collected and immediately placed on ice. It was transferred to CHLA within 24 h, where it was stored at −80 °C until its transport to the University of California, Davis for processing.

### 2.5. Method of Fecal Microbiota Transplantation

All rCDI subjects received fecal material from a universal donor. The rationale behind a universal donor was fourfold: to reduce cross-contamination, as family members and close acquaintances have a higher risk of exposure and infection with *C. difficile* [26]; to decrease confounding variables related to variability in the donor microbiome, allowing for greater generalizability among the subjects; to control for better outcomes [27]; and to streamline the process for ease and cost-efficiency.

The fecal material was prepared by an independent laboratory technologist according to the protocol described by Hamilton et al. [27]. Material from the donor was transported on ice to the laboratory where it was immediately processed and weighed. An amount of 50 g of stool was homogenized in a commercial blender in a biological cabinet and then passed through 2.0, 1.20, 0.5, and 0.25 stainless steel laboratory sieves. It was then centrifuged at 6000× *g* for 15 min, re-suspended in 250 mL of non-bacteriostatic normal saline, and immediately frozen at −70 °C. On the day of use, the fecal material was thawed in an ice bath over 2–4 h and immediately used.

### 2.6. Short-Chain Fatty Acid Quantification

We quantified the SCFAs butyrate, acetic acid, propionic acid, isovaleric acid, and formic acid using the methods that Rotondo et al. described in their paper [28]. We chose these specific SCFAs as these have been the ones that are studied extensively in the literature. Briefly, all analyses were conducted at the West Coast Metabolomics Center at the University of California, Davis. Here, 10 mg samples of feces were homogenized with 700 μL of water, hydrochloric acid, and methyl tert-butyl ether (5:1:1) using a Genogrinder at 1500 rpm for 30 s, shaken for 30 min at room temperature, then centrifuged for 2 min at 14,000 rcf. Then, 500 μL of the supernatant was transferred to a new tube, to which 0.1 g of anhydrous sodium sulfate was added for dehydration. An amount of 25 μL of N-tert-butyldimethylsilyl-N-methyltrifluoroacetamide (MTBSTFA, Sigma-Aldrich, St. Louis, MO, USA) was used for tert-butyldimethylsilylation. Samples were then shaken at 80 °C for 30 min. This solution was injected into an Agilent 5977A GC-quadrupole mass spectrometer, which was set to selected ion monitoring (SIM) mode. Raw data were processed using Agilent Mass Hunter Quantitative Analysis software (B.07.00). This was quantified against authentic standards [29].

### 2.7. Statistical Analysis

Participant demographics and clinical outcomes at baseline are described as raw data. Medians (IQR) were used to summarize participant SCFA levels overall and across groups. To efficiently assess the change between pre-transplant and post-transplant fecal SCFA levels, while taking advantage of repeat measures per individual, linear mixed-effects models with a random participant effect accounting for within-participant correlation were utilized. This analysis was conducted on each SCFA and on the combined mean of SCFA values to witness the mean change at the study timepoints (M1, M3, M6, M12) compared to pre-transplant (baseline) values. All models were adjusted for pre-transplant outcome (SCFA) values.

For our secondary aim, the difference in the value of fecal SCFA values at baseline between healthy controls and the pre-treatment rCDI cohort was assessed via Wilcoxon rank-sum tests. Understanding that the healthy control cohort is composed of older children, we performed a supplemental analysis excluding children over 11 years old in the healthy control cohort. Results from this analysis yield the same conclusions as the original analysis (Supplemental Table S1).

All tests were 2-sided, and *p*-values less than or equal to 0.05 were considered statistically significant, with no corrections for multiple hypothesis testing as the *p*-values generated were considered descriptive statistics. All statistical analyses were conducted by R-studio 4.2.2.

## 3. Results

An overview of the characteristics of rCDI FMT recipients and healthy controls are provided in Table 1 and Table 2, respectively. Additionally, our study population is compared in Table 3, Table 4 and Table 5. Though the control group had a higher mean age than the study group, there is evidence in the literature that SCFA concentrations stabilize in stool following the first year of life [30]. Following FMT, all recipients had clinical resolution of their CDI and were recurrence-free for up to 12 months, as confirmed by laboratory testing and the resolution of symptoms. There were no dietary changes reported in the rCDI group throughout the duration of their involvement in the study (up to 12 months post-FMT). All nine subjects provided pre- and at least one post-FMT stool sample. In addition, all nineteen healthy controls provided stool for comparison.

As seen in Table 4, the raw concentrations of fecal SCFA profiles display heterogeneity between the levels of the different SCFAs. This was expected, as acetic acid, butyric acid, and propionic acid have the highest concentration in the gut microbiome [31].

**Figure 1:**
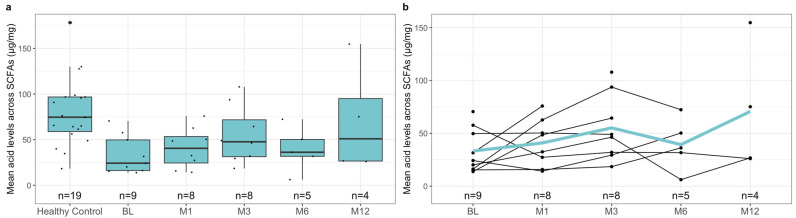
(**a**) Boxplots of fecal combined SCFA levels stratified by healthy control at baseline and *C. difficile* cohort at pre-treatment (BL), 1 month post-FMT (M1), 3 months post-FMT (M3), 6 months post-FMT (M6), and 12 months post-FMT (M12), with *n* represents the number of available data points. (**b**) Spaghetti plot of individual combined SCFA levels among *C. difficile* cohort at pre-treatment (BL), 1 month post-FMT (M1), 3 months post-FMT (M3), 6 months post-FMT (M6), and 12 months post-FMT (M12), with mean shown as the color line and *n* represents the number of available data points. The levels of acetic acid, butyric acid, formic acid, isovaleric acid, and propionic acid were quantified using mass spectroscopy, as described in Section 2, and are presented here in µg/mg. The difference in the levels of the sum of all SCFAs measured at BL between the healthy controls and rCDI patients, using the statistical tests described in Section 2, is statistically significant (*p* < 0.001). In addition, there was a significant overall increase in fecal SCFA levels in rCDI patients at 12 months post-FMT, when compared to BL (*p* = 0.015).

**Figure 2:**
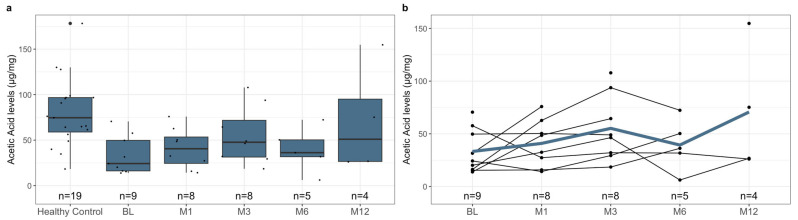
(**a**) Boxplots of fecal acetic acid levels stratified by healthy control at baseline and *C. difficile* cohort at pre-treatment (BL), 1 month post-FMT (M1), 3 months post-FMT (M3), 6 months post-FMT (M6), and 12 months post-FMT (M12), with *n* represents the number of available data points. (**b**) Spaghetti plot of individual fecal acetic acid levels among *C. difficile* cohort at pre-treatment (BL), 1 month post-FMT (M1), 3 months post-FMT (M3), 6 months post-FMT (M6), and 12 months post-FMT (M12), with mean shown as the color line and *n* represents the number of available data points. The acetic acid levels were quantified using mass spectroscopy, as described in Section 2, and are presented here in µg/mg. The difference in the levels of fecal acetic acid at BL between the healthy controls and rCDI patients, using the statistical tests described in Section 2, is statistically significant (*p* < 0.001). In addition, the improvement in fecal acetic acid levels in rCDI patients 12 months post-FMT, when compared to BL, is statistically significant (*p* = 0.039).

**Figure 3:**
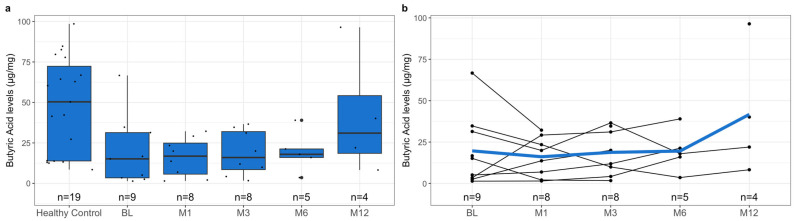
(**a**) Boxplots of fecal butyric acid levels stratified by healthy control at baseline and *C. difficile* cohort at pre-treatment (BL), 1 month post-FMT (M1), 3 months post-FMT (M3), 6 months post-FMT (M6), and 12 months post-FMT (M12), with *n* represents the number of available data points. (**b**) Spaghetti plot of individual fecal butyric acid levels among *C. difficile* cohort at pre-treatment (BL), 1 month post-FMT (M1), 3 months post-FMT (M3), 6 months post-FMT (M6), and 12 months post-FMT (M12), with mean shown as the color line and *n* represents the number of available data points. The butyric acid levels were quantified using mass spectroscopy, as described in Section 2, and are presented here in µg/mg. The difference in the levels of fecal butyric acid at BL between the healthy controls and rCDI patients, using the statistical tests described in Section 2, is statistically significant (*p* = 0.02). Although there was an improvement in butyric acid levels in rCDI patients post-FMT, the change was not statistically significant (*p* > 0.05).

**Figure 4:**
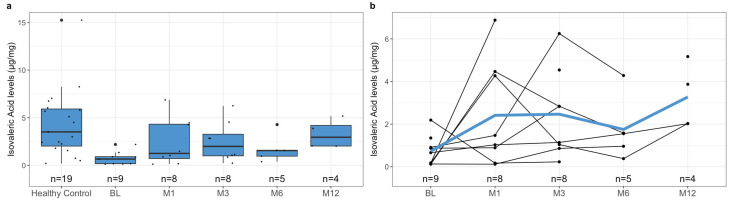
(**a**) Boxplots of fecal isovaleric acid levels stratified by healthy control at baseline and *C. difficile* cohort at pre-treatment (BL), 1 month post-FMT (M1), 3 months post-FMT (M3), 6 months post-FMT (M6), and 12 months post-FMT (M12). (**b**) Spaghetti plot of individual fecal isovaleric acid levels among *C. difficile* cohort at pre-treatment (BL), 1 month post-FMT (M1), 3 months post-FMT (M3), 6 months post-FMT (M6), and 12 months post-FMT (M12), with mean shown as the color line. The isovaleric acid levels were quantified using mass spectroscopy, as described in Section 2, and are presented here in µg/mg. The difference in the levels of fecal isovaleric acid at BL between the healthy controls and rCDI patients, using the statistical tests described in Section 2, is statistically significant (*p* < 0.001). Similarly, there is an improvement in fecal isovaleric acid levels witnessed in the rCDI cohort in response to FMT. The change from baseline is statistically significant at the following intervals: M1 (*p* = 0.04; 95% CI), M3 (*p* = 0.03; 95% CI), and M12 (*p* = 0.02; 95% CI).

**Figure 5:**
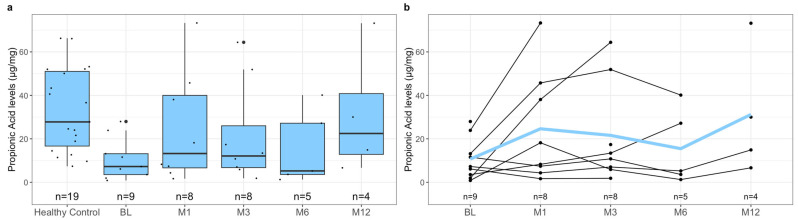
(**a**) Boxplots of fecal propionic acid levels stratified by healthy control at baseline and *C. difficile* cohort at pre-treatment (BL), 1 month post-FMT (M1), 3 months post-FMT (M3), 6 months post-FMT (M6), and 12 months post-FMT (M12), with *n* represents the number of available data points. (**b**) Spaghetti plot of individual fecal propionic acid levels among *C. difficile* cohort at pre-treatment (BL), 1 month post-FMT (M1), 3 months post-FMT (M3), 6 months post-FMT (M6), and 12 months post-FMT (M12), with mean shown as the color line and *n* represents the number of available data points. The propionic acid levels were quantified using mass spectroscopy, as described in Section 2, and are presented here in µg/mg. The difference in the levels of fecal propionic acid at BL between the healthy controls and rCDI patients, using the statistical tests described in Section 2, is statistically significant (*p* = 0.002). In addition, there was an improvement, though not statistically significant, in fecal propionic acid levels in rCDI patients at M1 (*p* = 0.05) and M3 (*p* = 0.06). The improvement in fecal propionic acid levels in rCDI patients 12 months post-FMT, when compared to BL, however, is statistically significant (*p* = 0.019; 95% CI).

**Figure 6:**
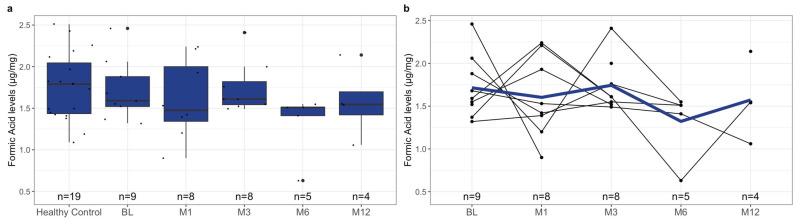
(**a**) Boxplots of fecal formic acid levels stratified by healthy control at baseline and *C. difficile* cohort at pre-treatment (BL), 1 month post-FMT (M1), 3 months post-FMT (M3), 6 months post-FMT (M6), and 12 months post-FMT (M12), with *n* represents the number of available data points. (**b**) Spaghetti plot of individual fecal formic acid levels among *C. difficile* cohort at pre-treatment (BL), 1 month post-FMT (M1), 3 months post-FMT (M3), 6 months post-FMT (M6), and 12 months post-FMT (M12), with mean shown as the color line and *n* represents the number of available data points. The formic acid levels were quantified using mass spectroscopy, as described in Section 2, and are presented here in µg/mg. The difference in the levels of fecal formic acid at BL between the healthy controls and rCDI patients, using the statistical tests described in Section 2, is not statistically significant (*p* = 0.80). In the cohort of rCDI patients, there was no statistically significant change from pretreatment values at any of the intervals (M1 *p* = 0.55, M3 *p* = 0.81, M6 *p* = 0.1, M12 *p* = 0.66).

To investigate the difference in pre-treatment SCFA levels between healthy controls and patients with rCDI, we measured the levels of acetic acid, butyric acid, formic acid, isovaleric acid, and propionic acid in both subgroups’ samples. Notably, the healthy controls had significantly higher mean fecal SCFA levels than the pre-treatment levels of pediatric patients with rCDI (Figure 1a, Table 4, *p*-value < 0.001). This relationship was reflected when comparing the individual SCFAs acetic acid (Figure 2a, Table 4, *p*-value < 0.001), butyric acid (Figure 3a, Table 4, *p*-value = 0.02), isovaleric acid (Figure 4a, Table 4, *p*-value < 0.001), and propionic acid (Figure 5a, Table 4, *p*-value = 0.002) of the two cohorts. Remarkably, formic acid was the only SCFA in which fecal levels were not statistically different between healthy and rCDI subjects (Figure 6a, Table 4, *p*-value = 0.80).

Given these changes between the two groups, we hypothesized that with the resolution of CDI, we would witness a restoration of SCFA levels in the rCDI cohort to resemble the healthy control profiles more closely. To assess this, we utilized a linear mixed model to examine the changes, when compared with pretreatment levels, in fecal SCFA levels at months 1, 3, 6, and 12 post-FMT. We observed a significant increase in overall fecal SCFA levels at month 12 when compared with pretreatment levels (Figure 1b, Table 6, *p*-value = 0.015). This was due to improvements in acetic acid, butyric acid, isovaleric acid, and propionic acid levels. The characterization of each of the fecal SCFA levels at the aforementioned intervals reveals that each SCFA had its unique pattern of recovery. The levels of isovaleric acid, for example, significantly increased from baseline at M1 (*p*-value = 0.04), M3 (*p*-value = 0.03), and M12 (*p*-value = 0.02) (Figure 4b, Table 6). In contrast, acetic acid and propionic acid levels were significantly increased from baseline only at M12 (*p*-value = 0.039, *p*-value = 0.019, respectively) (Figure 2b and Figure 6b, Table 6). Worthy to note, though the change in butyrate did not meet the 95% confidence interval, its levels did seem to increase during the increasing duration post-FMT, with month 12 having the most significant increase from baseline (Figure 3b, Table 6, *p*-value = 0.065).

## 4. Discussion

This study analyzes longitudinal SCFA levels up to 12 months post-FMT in pediatric patients who have recovered from rCDI. Both the longitudinal nature of the study and the results generated shed valuable light on the mechanism and timing of FMT in the treatment of pediatric patients with rCDI.

We demonstrated, by comparison with healthy controls within a similar age range, that decreased overall fecal SCFA levels and decreased individual levels of acetic acid, butyric acid, isovaleric acid, and propionic acid are associated with recurrent *C. difficile* colitis in the pediatric population. This was unsurprising, as a previous study has documented similar results in an adult population [32].

We also witnessed a recovery in SCFA levels from baseline to month 12 in our rCDI pediatric cohort, especially isovaleric acid and propionic acid at month 1. This is correlative with disease activity, as all subjects included in this study had no evidence of *C. difficile* colitis up to the study endpoint of 12 months, as well as clinical symptom resolution. Although prior literature has associated FMT success in rCDI humans with the restoration of SCFA levels [32,33], to our knowledge, only one has analyzed this change in humans longitudinally [32]. The aforementioned study’s follow-up period was limited to 6 months, though, and restricted to an adult population [32]. Therefore, this was the first study to analyze long-term (12 months) SCFA changes in *any* population. Moreso, there have been no prior studies of our knowledge that have studied longitudinal changes in SCFA levels post-transplant in pediatric rCDI patients in any capacity (short-term or long-term).

The recovery of acetic acid, butyric acid, isovaleric acid, and propionic acid levels have a variety of implications for the mechanisms by which the gut heals from *C. difficile*. Studies in the past have noted that the SCFAs—acetate, butyrate, propionate, and valerate—have a direct inhibitory role on the growth rate of *C. difficile* in culture [14,34]. In hamster models, a higher level of SCFAs is protective against *C. difficile* infection. This was consistent with the data that have proven that antibiotic use decreases SCFA levels, thereby suggesting that an SCFA-depleted gut can provide an environment favorable for *C. difficile* growth and germination [35]. Therefore, SCFAs are key to eradicating the pathogenic insult and preventing further disease.

Acetic acid, butyric acid, and propionic acid also have a variety of immune regulatory roles. They act as anti-inflammatory molecules through a variety of mechanisms: inhibiting the lipopolysaccharide-stimulated release of tumor necrosis factor α [24], inhibiting the NF-kB pathway [24], stimulating neutrophil chemotaxis [22], and regulating interleukin levels [36,37,38].

Higher acetic acid levels have been shown to decrease the permeability of the gut, thus ensuring the integrity of the mucosal epithelium [23]. Thus, the decreased pre-treatment levels of acetic acid in our rCDI cohort, combined with the fact that *C. difficile* toxins have been implicated in causing the disruption of epithelial paracellular barrier function [39], might explain a mechanism through which *C. difficile* pathogenesis occurs. Similarly, since we conclude that acetic acid levels increase in response to FMT, perhaps acetic acid is a key mediator of FMT’s anti-*C. difficile* action in the pediatric population.

In addition to its role in immunity, butyrate is an important metabolite in the gut lumen, being the main source of energy for the colonocyte [40]. It participates in a variety of roles in epithelial maintenance, including growth, differentiation, protection, and repair [41,42,43,44]. Therefore, it was unsurprising that we witnessed decreased levels in our rCDI population compared to healthy controls and an overall increase in butyric acid levels in the post-treatment era. Though month 12 butyrate levels were not significantly different by statistical definition, there was a dramatic increase in its level at month 12 compared to baseline than when compared to other time intervals (*p*-value = 0.065). There was, however, a significant difference when comparing butyrate levels in pediatric patients with rCDI at baseline with healthy controls. The dichotomy between a non-significant increase in butyrate levels at post-FMT month-12 follow-up and a significant difference in butyrate levels at baseline could be due to a few factors, such as sampling size, or that there might be more follow-up time necessary to witness full butyrate level restoration.

Unlike polysaccharide-derived metabolites such as acetate, butyrate, and propionic acid, isovaleric acid is a product of amino acid fermentation and is a branched-chain fatty acid. Compared to other metabolites, prior research on isovaleric acid and its involvement in gut homeostasis is scarce. Thus, its role in the gut is vague, though there is evidence that it can lead to smooth muscle relaxation in the gut lumen through the cAMP/PKA pathway [45]. Additionally, isovalerate has been found to potentially increase SUMOylation, leading to the inhibition of inflammation through the NF-kB pathway and preserving intestinal cell function and integrity [46]. Though there is limited research, we would like to further explore the importance of isovaleric acid in microbiota metabolism, mucosal healing, and immune response in a future study.

The longitudinal nature of this study is significant, as SCFA profiles were only significant when examining the change from baseline to month 12. This is consistent with prior research that suggests that there are post-FMT fecal microbiota changes that occur, although this study was shorter in duration and explored an older population [47].

We readily acknowledge the limitations of our study. First, due to the lack of patient adherence to longitudinal stool collections, the statistical power of our longitudinal data decreases over the study duration. Four of the nine children had no available stool at month six, and five of the nine had no stool at the final visit. As a result, our month-12 data only represent the SCFA profiles of four patients and thus may not be generalizable to a larger population. Additionally, the limited data may have influenced our recorded findings. Second, there is evidence that even short-term fluctuations in diet can impact the composition and metabolism of the microbiome, affecting overall SCFA composition [48]. Though no dietary changes were reported in our population, we cannot be sure that day-to-day variance was without influence on our data, especially given our small cohort size. Third, there was no age stratification qualifier in our control group due to suggestive evidence that SCFA profiles are in stabilized concentrations after the first year of life [30]. Additionally, we ran a supplemental analysis that excluded children above 11 years old and this yielded similar conclusions as the literature. It is evident that the healthy controls had much higher SCFA levels than the study population, suggesting age to be an insignificant confounding factor.

## 5. Conclusions

We identified an inverse association between pediatric recurrent *C. difficile* infection and fecal levels of short-chain fatty acids. This relationship was demonstrated both in comparison with healthy controls at baseline, as well as with the restoration of SCFA levels 12 months after FMT in the rCDI cohort. In particular, acetic acid, butyric acid, propionic acid, and isovaleric acid levels increase after therapy, though the levels of butyric acid did not meet the threshold of statistical significance.

## Figures and Tables

**Table 1:** rCDI FMT recipient characteristics.

Subject Number	Age at Transplant (Years)	Biological Sex	Comorbidities	Current Medication	Number of Prior *C. difficile* Infections	BMI Percentile	Ethnicity
1	6	Female	Developmental delay, chronic lung disease, gastrostomy status	PPI, erythromycin ^a^	>5	13	Caucasian
2	2	Male	Cleft lip, GERD	None	>5	37	Latino
3	7	Male	Developmental delay, gastrostomy status	Erythromycin ^a^	>5	21	Latino
4	10	Female	None	None	3	46	Caucasian
5	3	Female	Developmental delay, recurrent pneumonia	PPI	>5	54	Caucasian
6	9	Male	ASD	None	3	33	Caucasian
7	17	Male	Developmental delay, gastrostomy status	PPI	4	51	Asian
8	12	Male	None	None	>5	62	Caucasian
9	4	Female	SBS	Iron, Multivitamin	>5	5	Caucasian

Abbreviations: PPI, proton-pump inhibitor; FMT, fecal microbiota transplantation; GERD, gastroesophageal reflux disorder; ASD, autism spectrum disorder; SBS, short bowel syndrome; ^a^ the erythromycin used in these 2 subjects was for prokinetic effect (small doses) and not antimicrobial dosing.

**Table 2:** Healthy control characteristics.

Healthy Control Number	Age at Study Visit (Years)	Biological Sex	BMI Percentile
1	14	Female	51
2	12	Female	12
3	14	Male	65
4	14	Female	6
5	12	Female	48
6	9	Female	15
7	9	Female	8
8	12	Male	79
9	14	Male	80
10	14	Female	19
11	15	Female	14
12	10	Male	82
13	11	Female	50
14	11	Female	25
15	16	Male	17
16	13	Male	8
17	10	Male	54
18	15	Male	20
19	2	Male	52

Healthy controls have no active illnesses nor concurrent medication use at the time of inclusion. Of the 19 healthy controls, 17 are Caucasian, 1 is African American, and 1 is Asian.

**Table 3:** Population study demographics.

Characteristic	Healthy Controls	*C. difficile* Patients
Age Average (years)	11.95 (range: 2–16)	7.78 (range: 2–17)
Gender (Male:Female)	9:10	5:4
BMI Percentile Average	37.11 (range: 6–82)	35.78 (range: 5–62)

The average age, male/female ratio, and BMI percentile average were compared between the healthy controls and the *C. difficile* patients.

**Table 4:** SCFA profile comparison between healthy controls and baseline.

Characteristic	Overall, N = 28	*C. difficile,* N = 9	Healthy Control, N = 19	*p*-Value
Mean across all SCFAs	23.47 (14.14, 38.49)	11.46 (7.11, 19.73)	35.67 (21.76, 42.45)	<0.001
Acetic acid	62.87 (33.97, 92.07)	24.25 (16.18, 49.72)	74.60 (58.91, 96.78)	<0.001
Butyric acid	33.06 (13.16, 64.95)	15.15 (3.43, 31.37)	50.37 (13.87, 72.33)	0.02
Formic acid	1.71 (1.44, 1.99)	1.59 (1.52, 1.88)	1.79 (1.44, 2.04)	0.8
Isovaleric acid	2.22 (0.72, 5.25)	0.66 (0.17, 0.91)	3.51 (2.02, 5.92)	<0.001
Propionic acid	22.75 (10.97, 41.28)	7.25 (3.54, 13.15)	27.79 (16.67, 51.05)	0.002

Values are presented as median (IQR). The difference in fecal SCFA values at baseline between healthy controls and the pre-treatment rCDI cohort was assessed via Wilcoxon rank-sum tests.

**Table 5:** Donor stool SCFA profile comparison to healthy controls.

Compound	Donor Stool (µg/mg)	Healthy Control Stool (µg/mg)
Acetic Acid	58.05	99.26
Butyric Acid	68.48	60.22
Formic Acid	1.73	2.10
Isovaleric Acid	3.73	3.36
Propionic Acid	34.29	50.32

The SCFA profiles between the donor and the healthy stools were compared, with the profiles being very similar.

**Table 6:** Assessment of changes from baseline in SCFA levels in *C. difficile* patients at study timepoints (M1, M3, M6, M12).

Time Point	All SCFAs		Acetic Acid		Butyric Acid		Formic Acid		Isovaleric Acid		Propionic Acid	
	Mean Change	*p* Value	Mean Change	*p* Value	Mean Change	*p* Value	Mean Change	*p* Value	Mean Change	*p* Value	Mean Change	*p* Value
Month 1	4.7 (−5.4, 14.7)	0.36	10.5 (−13.7, 34)	0.42	−3.54 (−20.2, 13)	0.69	−0.12 (−0.5, 0.2)	0.55	1.74 (0.2, 3.2)	0.04	14.53 (1.7, 27.5)	0.05
Month 3	8.06 (−2.2, 18)	0.12	22.77	0.09	1.45 (−15.8 17.8)	0.87	0.05 (−0.3, 0.4)	0.81	1.83 (0.3, 3.3)	0.03	14.02 (0.9, 26.8)	0.06
Month 6	6.3 (−6.1, 17.8)	0.3	15.21 (−14.6, 42.4)	0.32	2.62 (−17.8, 21.4)	0.8	−0.38 (−0.8, 0)	0.1	1.25 (−0.6, 2.9)	0.19	11.15 (−4.3, 26.2)	0.18
Month 12	16.24 (3.5 29)	0.015	35.46 (5.4, 65.8)	0.039	21.47 (0.9, 42.1)	0.065	−0.11 (−0.6, 0.3)	0.656	2.51 (0.6, 4.4)	0.02	22.51 (5.9, 38.9)	0.019

Assessment of change from baseline in longitudinal samples adjusted for pre-transplant outcome (SCFA) values. All values are presented as mean change from baseline (95% confidence interval). All SCFA levels were measured in µg/mg.

## Data Availability

The data presented in this study are available on request from the corresponding author. The data are not publicly available due to privacy.

## Supplementary Materials

The following supporting information can be downloaded at: https://www.mdpi.com/article/10.3390/metabo13101039/s1, Supplemental Table S1: Age and SCFA profile comparison between healthy controls and baseline (excludes participants over 11 years old in the healthy controls).
