# Supplementary material for: Short-Chain Fatty Acid Levels after Fecal Microbiota Transplantation in a Pediatric Cohort with Recurrent Clostridioides difficile Infection
Source: Metabolites. 2023 Sep 27;13(10):1039. doi: 10.3390/metabo13101039 (PMC10608736; doi:10.3390/metabo13101039)
Supplement: Supplementary file 1 [file metabolites-13-01039-s001.zip › metabolites-2589250-supplementary.pdf]

Supplemental Table S1: Age and SCFA Profile Comparison Between Healthy Controls & Baseline (Excludes participants over 11 years old in the healthy controls)

Characteristic	Overall, N =16	C. *difficile,* N = 9	Healthy Control, N =7	p - value
Age (years)	8.25 (4.06)	7.78 (4.79)	8.86 (3.13)	0.5
Mean across all SCFA's	20.64 (10.39, 25.10)	11.46 (7.11, 19.73)	25.55 (22.43, 40.68)	0.008
Acetic Acid	56.98 (23.23, 71.97)	24.25 (16.18, 49.72)	76.19 (64.89, 93.82)	0.002
Butyric Acid	21.97 (11.59, 41.63)	15.15 (3.43, 31.37)	41.45 (20.60, 51.27)	0.11
Formic Acid	1.71 (1.52, 1.93)	1.59 (1.52, 1.88)	1.79 (1.62, 1.97)	0.5
Isovaleric Acid	1.13 (0.19, 3.13)	0.66 (0.17, 0.91)	3.51 (2.40, 5.10)	0.005
Propionic Acid	13.80 (6.97, 24.91)	7.25 (3.54, 13.15)	21.60 (16.67, 52.08)	0.023

Supplementary Table S1: SCFA Profile Comparison Between Healthy Controls & Baseline (exclude participants over 11 years old in the healthy controls). Values are presented as mean (SD) and median (IQR). Difference in the value of fecal SCFA values at baseline between healthy controls and the pre-treatment rCDI cohort was assessed via Wilcoxon rank-sum tests.
